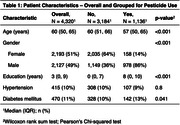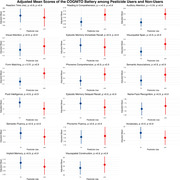# Association of Pesticide Use with Cognition in Aging Community‐Dwelling Rural Adults in South India

**DOI:** 10.1002/alz.091475

**Published:** 2025-01-09

**Authors:** Suhrud Panchawagh, Sumedha Mitra, Jonas S. Sundarakumar

**Affiliations:** ^1^ Smt. Kashibai Navale Medical College & General Hospital, Pune, Maharashtra India; ^2^ Centre for Brain Research, Indian Institute of Science, Bangalore, Karnataka India

## Abstract

**Background:**

Emerging evidence suggests environmental factors, such as chronic pesticide exposure, may influence cognitive functioning, yet research in this area remains limited, particularly in rural Indian settings. Our objective was to investigate the potential link between pesticide exposure and cognitive performance in an aging population from rural India.

**Method:**

This cross‐sectional analysis included 4320 participants from the Srinivasapura Aging NeuroSenescence and COGnition (SANSCOG) cohort. They are predominantly farmers, recruited from the rural areas of Srinivaspura in southern India. Pesticide exposure was assessed based on self‐reporting of usage. Cognitive performance was evaluated using a comprehensive, culturally adapted, computerized neurocognitive battery (COGNITO), which encompasses various domains of cognitive function, such as attention (reaction time, visual attention, auditory attention), memory (immediate and delayed episodic memory, implicit memory, name‐face recognition), language (reading & comprehension, phoneme comprehension, phonemic fluency, semantic associations, vocabulary), visuospatial ability (visuospatial span and visuospatial construction), and executive function (form matching, fluid intelligence, semantic fluency). We used multivariate regression models, adjusting for potential confounders such as age, gender, education, diabetes mellitus, and hypertension to test the association between pesticide use and cognition. Multiple comparisons were handled using the Bonferroni correction.

**Result:**

In our study, 3661/4320 participants (85%) were involved in agriculture, of which 1136/3661 (31%) used pesticides. (Table 1) The median duration of pesticide usage was 10 years (IQR: 5, 15). Our study did not find a statistically significant association between high pesticide use and cognitive performance scores in any domain after adjusting for multiple comparisons. (Figure 1)

**Conclusion:**

Our analysis reports that moderate‐term pesticide use did not significantly impact cognitive function in this population of aging rural Indian adults. However, the cross‐sectional nature, lack of objective information on the severity of exposure, and potential confounders suggest caution in interpretation. We intend to follow up these participants with periodic cognitive monitoring to examine if long‐term pesticide exposure could be linked to accelerated cognitive decline and increased risk for dementia.